# Kynurenine‐3‐monooxygenase: A new direction for the treatment in different diseases

**DOI:** 10.1002/fsn3.1418

**Published:** 2020-01-20

**Authors:** Yifei Lu, Mingmei Shao, Tao Wu

**Affiliations:** ^1^ Institute of Interdisciplinary Integrative Medicine Research Shanghai University of Traditional Chinese Medicine Shanghai China

**Keywords:** KMO, kynurenic acid, kynurenine pathway, mechanism, quinolinic acid

## Abstract

Kynurenine‐3‐monooxygenase (KMO) is an enzyme that relies on nicotinamide adenine dinucleotide phosphate (NADP), a key site in the kynurenine pathway (KP), which has great effects on neurological diseases, cancer, and peripheral inflammation. This review mainly pay attention to the research of KMO mechanism for the treatment of different diseases, and hopes to provide assistance for clinical and drug use. KMO controlling the chief division of the KP, which directly controls downstream product quinolinic acid (QUIN) and indirectly controls kynurenic acid (KYNA), plays an important role in many diseases, especially neurological diseases.

## Graphical abstract

Kynurenine‐3‐monooxygenase (KMO) plays a very important role on the way of tryptophan catabolism through kynurenine pathway and has a definite regulatory effect on peripheral inflammation and central nervous system diseases. This review provides a systematic overview of KMO's research on the treatment mechanism of different diseases.

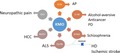

## 1 INTRODUCTION

**Figure 1:**
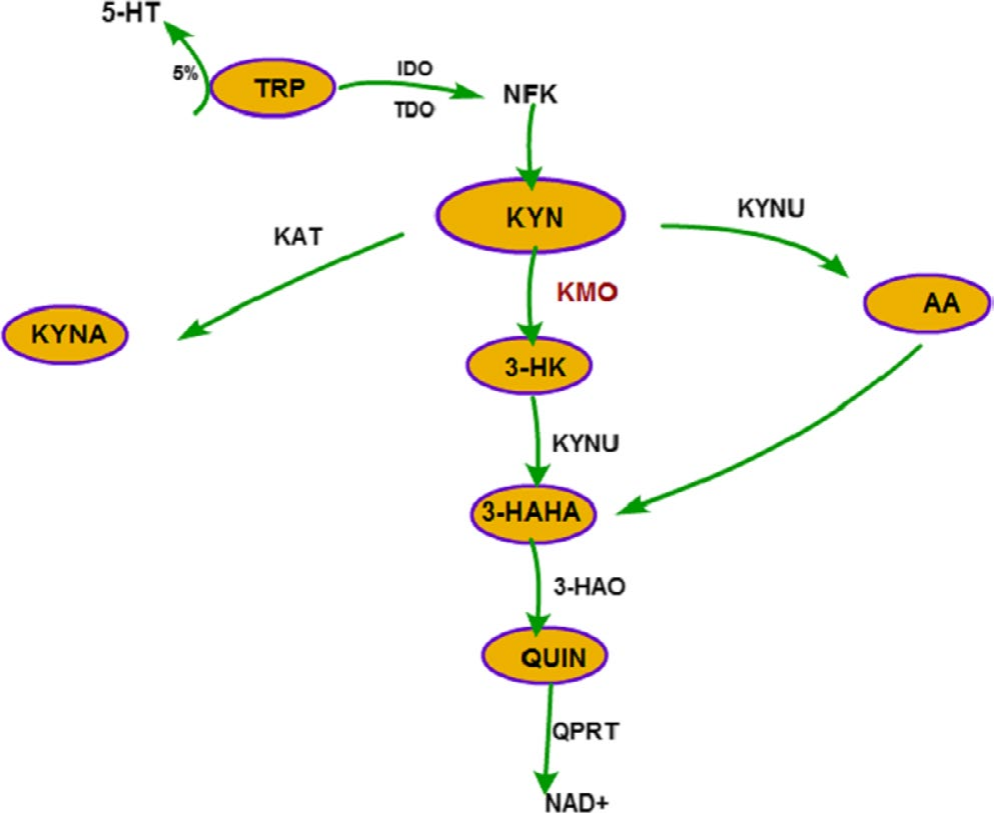
The kynurenine pathway. 3‐HANA, 3‐hydroxyl anthranilic acid; 3‐HAO, 3‐hydroxyanthranolic acid dioxygenase; 3‐HK, 3‐hydroxykynurenine; AA, anthranilic acid; IDO, indole‐2,3‐dioxygenase; KAT, kynurenine aminotransferase; KMO, kynurenine‐3‐monooxygenase; KYN, kynurenine; KYNA, kynurenic acid; KYNU, kynurenase; NAD+, nicotinamide adenine dinucleotide; NFK; N‐Formylkynurenine; QPRT, quinolinate phosphoribosyl transferase; QUIN, quinolinic acid; TDO, tryptophan‐2,3‐dioxygenase; TRY, tryptophan

Kynurenine‐3‐monooxygenase (KMO) is part of the three enzymes in the KP contained flavin adenine dinucleotide (FAD) (Smith, Jamie, & Guillemin, 2016) which was used for catalyzing the conversion of l‐kynurenine to 3‐hydroxykynurenine and water. It was revealed that kidney and liver had the most enzyme activity while least activity was found in brain (Erickson, Flanagan, Russo, & Reinhard, 1992). However, effects of oxygen as substrate from brain and liver KMO activity were nearly alike (Dang, Dale, & Brown, 2000). The KP is the principal pathway for the metabolism of tryptophan (TRY). About 5% tryptophan synthesizes serotonin (5‐HT) in intestinal chromaffin cells, and most of TRY is metabolized by KP to kynurenine (KYN) in peripheral tissues (such as liver and kidney) and central nervous system (e.g., astrocytes, microglia). As shown in Figure 1, TRP firstly produces N‐Formylkynurenine (NFK) under the catalysis of indole‐2,3‐dioxygenase (IDO) or tryptophan‐2,3‐dioxygenase (TDO) and then produces KYN under the catalysis of formamidase. KYN has three metabolic pathways. The first pathway, under the action of KMO, kynurenase (KYNU), and 3‐hydroxyanthranolic acid dioxygenase (3‐HAO), sequentially produces 3‐hydroxykynurenine (3‐HK), 3‐hydroxyl anthranilic acid (3‐HANA) and QUIN. QUIN produces the final metabolite nicotinamide adenine dinucleotide (NAD+) of KP catalyzed by quinolinate phosphoribosyl transferase (QPRT). In the second pathway, KYN produces kynurenine acid in the role of kynurenine aminotransferase (KAT), and on the other pathway, KYN produces anthranilic acid (AA) catalyzed by KYNU (Figure 1). On the way of tryptophan catabolism and the high activity of KMO in liver and kidney (Bertazzo, Ragazzi, & Biasiolo, 2001), KMO plays a very important role and has a definite regulatory effect on peripheral inflammation (Wilson et al., 2016) and central nervous system diseases. With more attention on the potential therapeutic effects of KMO, therefore, the present article provides a systematic overview of KMO's research on the treatment mechanism of different diseases.

## 2 THE STRUCTURE AND FUNCTION OF KMO

Studies on the structure and mechanism of KMO in animal (Zhang et al., 2018) have been discussed in the past (Kim et al., 2018; Smith et al., 2016). KMO is situated in the outer membrane of mitochondria (Quan et al., 2002) as a membrane‐associated protein (Gao et al., 2018), and its crystal structure was found by Amaral et al. (2013) as well as the first successful bacterial (*Escherichia coli*) expression of active human KMO enzyme expressed in the soluble fraction found by Wilson et al. (2014). A relative gauge of KMO activity by using mass spectrometry‐multiple‐reaction monitoring (MS‐MRM) (Winkler et al., 2013) and RapidFire mass spectrometry (RF‐MS) (Lowe et al., 2014) was detected and a more exact and effective screening of this class of enzymes in multiple assay formats was permitted. Because KMO inhibitors are difficult to pass through the blood–brain barrier, a facile fluorescence‐based KMO activity assay was reported by Jacobs, Guillemin, and Lovejoy (2018). KMO is a solitary gene and is composed of ten exons distributed over a 16‐kb region (Quan et al., 2002) which encoding that contains a FAD (noncovalent bond, but tightly bound) binding domain. It is a nicotinamide adenine dinucleotide phosphate (NADP)‐dependent flavin protease with the oxidation of l‐kynurenine to 3‐hydroxykynurenine by its catalysis, which consumes nicotinamide adenine dinucleotide phosphate (NADPH) and oxygen per conversion a molecule (Dang et al., 2000). KMO controls the synthesis of several metabolites of KP, including 3‐HK, QUIN, KYNA, and AA. Moreover, KMO controls the KP through both upstream and downstream (Wilson et al., 2016). The most influential are KYNA and QUIN (Giorgini et al., 2013) because they can affect excitatory glutamate signaling by binding to N‐methyl‐d‐aspartic acid receptor (NMDAR). It was shown that KMO appeared more active and guides the transformation of tryptophan to 3‐HK (Bertazzo et al., 2001) which have neurotoxic effects, the same as QUIN. It has an important regulating effect on nervous system diseases. But the neuroprotection of KMO inhibition through accumulation of KYNA which has neuroprotective effects and resulting attenuation of NMDA receptor function remains to be ascertained (Urenjak & Obrenovitch, 2000). At the same time, the resistance to 3‐HK‐mediated cell stress was affected by KMO overexpression (Wilson et al., 2016). Flaviano Giorgini et al. (2013) found that compared with control mice, the downstream metabolite QUIN in the KP pathway was significantly reduced in liver and plasma and slightly decreased in the brain in mice which KMO was knocked out, while extracellular urinary QUIN was significantly elevated. Therefore, KMO has a significant effect on the kynurenine metabolism pathway.

## 3 THE MECHANISM OF KMO IN DIFFERENT DISEASES

**Figure 2:**
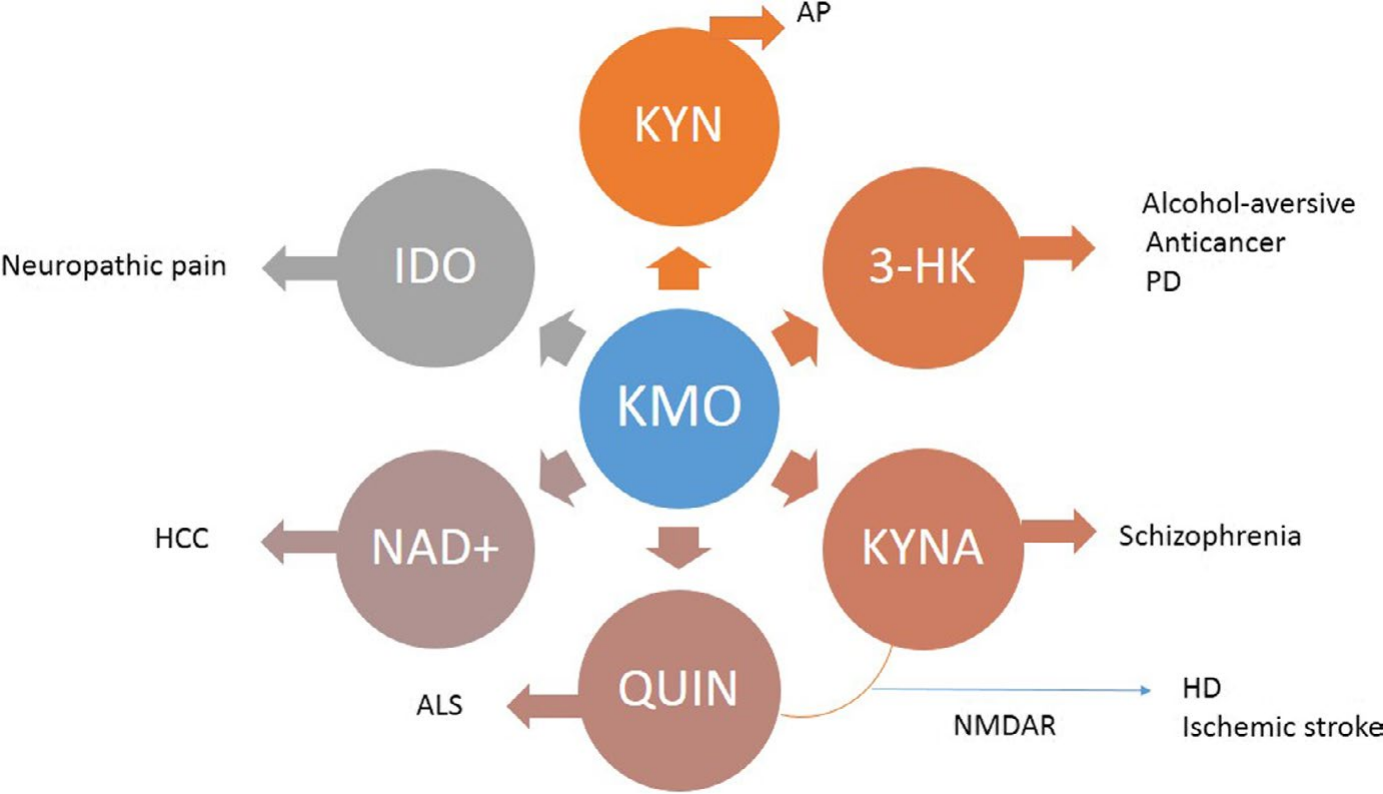
The role of KMO in different diseases. 3‐HK, 3‐hydroxykynurenine; ALS, amyotrophic lateral sclerosis; AP, acute pancreatitis; HCC, hepatocellular carcinoma; HD, Huntington's disease; IDO, indole‐2,3‐dioxygenase; KMO, kynurenine‐3‐monooxygenase; KYN; kynurenine; KYNA, kynurenic acid; NAD+, nicotinamide adenine dinucleotide; NMDAR, N‐methyl‐d‐aspartic acid receptor; PD, Parkinson's disease; QUIN, quinolinic acid

According to the different distributions of KMO in vivo, different mechanisms of action on different diseases through KMO were shown in Figure 2.

### 3.1 Nervous system diseases

KMO is mainly found in microglia in the central nervous system. In innate physiology, KP participates in the regulation of early brain development (Forrest et al., 2015). KP facilitates the formation of KYNA in the brain, and physiologically, QUIN‐induced neurodegeneration is blocked by KYNA (Zwilling et al., 2011). However, the disruption of homeostasis may shift the balance to increase QUIN (Parrott & O'Connor, 2015). Many studies have shown that the disorder of KP is associated with various neurological diseases, for example Alzheimer's disease, Huntington's disease, schizophrenia, ischemic stroke, Parkinson's disease, amyotrophic lateral sclerosis, and neuropathic pain (Rojewska, Piotrowska, Makuch, Przewlocka, & Mika, 2016). The research indicates that KMO can directly control the production of QUIN which is an N‐methyl‐d‐aspartate (NMDA) receptor agonist and indirectly control the production of kynurenate which is an NMDA receptor antagonist (Crozier & Moran, 2007). It was revealed that AA was much more readily converted into 3‐HANA in the brain (Maddison & Giorgini, 2015). When the immune system is activated, KMO is stimulated by cytokines in the periphery and brain, which activates inflammation‐mediated dysregulation of the KP, producing neurogenic excitatory damage, which is responsible for many major brain diseases (Garrison et al., 2018), and the lower KMO expression and the higher KYNA production may contribute to dysfunctional effector CD4+ T‐cell response (Rad Pour et al., 2019). In addition, KMO gets immune in neurons and astrocytes of most of the forebrain and spinal cord regions (Chiarugi, Cozzi, Ballerini, Massacesi, & Moroni, 2001). The massive accumulation of QUIN in the brain activates NMDA receptors in nerve cells and astroglia to produce excitotoxicity and cause lipid peroxidation and increase oxidative stress. On the other hand, KYNA synthesis in the brain can be increased by KMO inhibitors while glutamate extracellular concentration in the basal ganglia was selectively reduced (Moroni et al., 2005) and endogenous KYNA preferentially controls the vulnerability of striatal neurons to QUIN (Sapko et al., 2006). In the disorder of KP, the researchers also found that a systemic inflammatory induced by IDO expression was not related to interferon‐γ (IFN‐γ) production, and KAT expression was much higher than KMO expression which may cause acute inflammation‐induced the induction of KMO neurotoxicity (Connor, Starr, O'Sullivan, & Harkin, 2008). Recently, some researchers find that KMO implicates neurons biphasically which are extremely subtle to both oxidative stress and energy deprivation. This may provide new therapeutic strategies to treat neurodegenerative disease (Castellano‐Gonzalez et al., 2019). Acute activation of KMO promotes the synthesis of NAD+ while production of reactive oxygen species (ROS), mitochondrial damage and reduced spare‐respiratory capacity (SRC) was induced by chronic KMO activation. The result of the vicious cycle is that the QUIN produced by the KMO branch of KP becomes the most important factor for mitochondrial disorders (Castellano‐Gonzalez et al., 2019). In addition to the function of KMO in the brain, the researcher also found that neuroprotection is conferred sufficiently through that tryptophan metabolite kynurenine accumulates and actively transports from the periphery to the brain, which is then converted to KYNA (Zwilling et al., 2011). In conclusion, the role of KMO in the treatment of neurological diseases is mainly influenced by its directly controlled QUIN and indirectly controlled KYNA; thus, the study of their relationship may be a direction for future treatment and KMO inhibitors may be the sustained therapeutic potential in nervous system diseases (Pellicciari et al., 2003; Richter & Hamann, 2003) and prefer the neuroprotective function (Amori, Guidetti, Pellicciari, Kajii, & Schwarcz, 2009).

#### 3.1.1 Huntington's disease (HD)

Huntington's disease (HD) is a fatal neurodegenerative disease caused by the increase of the polyglutamine bundle in Huntington's protein (HTT), resulting in its accumulation in nuclear and cytoplasmic inclusions. Korrapati Sathyasaikumar et al. (2010) suggested that elevated 3‐HK in Huntington mouse was possibly due to the increased KMO activity. Also, many KMO inhibitors have shown therapeutic effects. Giorgini, Guidetti, Nguyen, Bennett, and Muchowski (2005) found Bna4 (kynurenine 3‐monooxygenase) can most effectively inhibit the toxicity of yeast gene mutation HTT fragment, which is straightly associated with the pathophysiology of Huntington's disease. Campesan et al. (2011) found that either pharmacological or genetic inhibition of KMO can dramatically reduce neurodegeneration of rhabdomeres in fruit fly model. Zwilling et al. (2011) found that JM6, a new small molecule prodrug inhibitor of KMO, reduced the activation of microglia in the Huntington's mouse model. Beaumont et al. (2016) found that KMO inhibitor can inhibit the formation of 3‐HK and QUIN, as well as raised levels of KYN and KYNA in brain tissue. This indicates that KMO has a very important role in KP. This has become a modern candidate for the therapy of Huntington's disease. Nevertheless, some researchers found that behavioral phenotypes or natural progression in HD cannot be modified significantly by the chronic closing of a selective KMO inhibitor (Beaumont et al., 2016). Together, KMO is still an essential enzyme in the therapy but needs more in‐depth research.

#### 3.1.2 Schizophrenia

Patients with schizophrenia display a significant reduction in KMO gene expression (Wonodi et al., 2011), and an increase in brain and cerebrospinal fluid (CSF) concentrations of the endogenous N‐methyl‐d‐aspartate receptor antagonist. KYNA, a terminal metabolite of the KP, may related to KAT enzyme activity via increased mRNA in astroglia by proinflammatory‐driven increase (Kindler et al., 2019) and its formation also indirectly depends on the action of KMO. Ikwunga Wonodi et al. (2011) found that the decrease in KMO activity may be one of the pathogenesis that related neurocognitive deficits and schizophrenia. Aoyama et al. (2006) believed that the metabolic changes of the KYNA pathway were associated with the etiology of schizophrenia. It is known that KMO inhibitors rise KYNA levels, and the KMO gene is positioned in the chromosomal region related to schizophrenia, 1q42‐q44. This suggests that KMO is a reliable candidate gene for schizophrenia (Erhardt et al., 2017). KMO has a critical effect on the pathophysiology of schizophrenia (Beggiato, Notarangelo, Sathyasaikumar, Giorgini, & Schwarcz, 2018; Reus et al., 2018). On the other hand, some researchers did not find a significant association between the KMO gene polymorphisms and the susceptibility to schizophrenia (Aoyama et al., 2006). However, support for KMO polymorphism does not confer primary susceptibility to schizophrenia itself (Holtze et al., 2011). At this point, some researchers also found that major susceptibility to schizophrenia is not conferred by KMO single nucleotide polymorphisms (SNPs) per se (Holtze et al., 2012). At the same time, Sathyasaikumar et al. (2011) suggested that the normalization of cortical KP metabolism through a persistent reduction in KMO activity shifting KP toward enhanced KYNA synthesis. Altogether, the inhibition of KMO treatment of schizophrenia is clear and effective, but its peculiar susceptibility is the focus of research.

#### 3.1.3 Ischemic stroke and neuropathic headache

KMO directly controls the production of quinolinate and NMDA receptor agonists (Crozier & Moran, 2007), and inhibition of KMO alters the equilibrium of these molecules (Crozier & Moran, 2007), thereby reducing the cell concentration of quinolinate and enhancing the cellular concentration of kynurenine. The effect of these NMDA receptor effectors makes KMO become an attractive target for the treatment of ischemic stroke (Crozier & Moran, 2007). In addition, Rojewska et al. (2016) also found that in the neuropathic pain model, inhibition of KMO function can dramatically reduce pain symptoms and enhance the effectiveness of morphine. But interestingly, Laumet et al. (2017) found that pain was independent of activation of neuromal KMO. Moreover, Rojewska, Ciapala, Piotrowska, Makuch, and Mika (2018) found that IDO2 and KMO may be the new treating targets for neuropathic pain. Nagy‐Grocz et al. (2017) also found that the mechanism of nitroglycerin on treating primary headache may be related to KP regulated by KMO. NMDAR is an important intermediate substrate for KMO treatment of ischemic stroke, and the upstream product IDO of KMO is also a problem worthy of study.

#### 3.1.4 Alzheimer's disease

Alzheimer's disease (AD), also called senile dementia and the most common form of irreversible dementia (Citron, 2010), is a degenerative, progressive neurological degenerative disease. The deposition of β‐amyloid protein (Aβ) in senile plaques and small arteries in the brain parenchyma plays a critical role in the pathogenesis of AD (Citron, 2010). In the same way, plasma neurofilament light (NFL) has been proved as a blood‐based biomarker for AD specificity especially in cognitive decline (Lin, Lee, Wang, & Fuh, 2018). Recently, the relevance between above two markers (NFL and Aβ) and KP metabolites has proposed that NFL may positively correlate with KP metabolites irrespective of NFL status while plasma Aβ seems to be NAL status dependent (Chatterjee et al., 2019). Therefore, interventions in the amyloid pathway are still the focus of most drug discovery efforts and seem to be safe at least some of these treatments (Citron, 2010). However, whether it is effective or not is still unknown. KP plays an important part in neurodegenerative disorders including Alzheimer's disease (Chouraki et al., 2017; Giil et al., 2017). Studies have shown that AD patients suffered with increased tryptophan through significantly decreased KYNA (Hartai et al., 2007) and increased level of QUIN (Gulaj, Pawlak, Bien, & Pawlak, 2010). Recently, Zwilling et al. (2011) proved that the use of non‐blood–brain barrier (BBB) penetrating KMO inhibitors in AD mouse models obviously improved symptoms and slowed disease progression. Jacobs et al. (2019) also suggested that peripheral plasma KP metabolites may be significantly associated with Aβ, total tau, and phosphorylated‐tau very likely through KMO regulation. It may be a new diagnostic method for AD with the advantage of its simple and less harmful. Research on KMO treatment of Alzheimer's disease is still in its infancy, and the mechanism research is still vague.

#### 3.1.5 Parkinson's disease (PD)

The main pathological change of Parkinson's disease is the degeneration of DA neurons in dense part of substantia nigra and Lewy body in the cytoplasm of the remaining neurons. Disorder of interactions between dopamine and glutamate with striatum DA content decreased, the acetylcholine (Ach), 5‐HT, and NE transmitters unbalanced is altered in Parkinsonism, resulting in an upregulation of corticostriatal glutamatergic function (Samadi et al., 2005). The content of 5‐HT, KYN, and KYNA in the prefrontal cortex nucleus and substantia nigra parsing of PD patients was low, while the concentration of 3‐HK was increased, suggesting that toxic products in KP may be involved in PD pathogenesis (Chang et al., 2018). The elevation of KYNA levels through inhibition of KMO may be a promising therapy for PD (Samadi et al., 2005).

#### 3.1.6 Amyotrophic lateral sclerosis

Amyotrophic lateral sclerosis (ALS) is a progressive and fatal motor neuron disease of unknown pathogenesis, with increased levels of CSF and serum QUIN (Chen, Brew, & Guillemin, 2011). Studies have shown that KP would be more beneficial in the short term of ALS and become detrimental in the long term (Lim, Brew, Sundaram, & Guillemin, 2010). This suggests that during neural inflammation, the disorder of KP may be a contributing factor in ALS (Chen et al., 2010; Lee et al., 2017) and some cure such as wedelolactone (WL) protecting the neurons from toxicity caused by QUIN through KP dysregulation (Maya, Prakash, & Goli, 2018) has been shown to work.

### 3.2 Non‐neurological disease

KMO is extensively scattered in non‐neural tissues, for example, liver, kidney, macrophages, microglia, and monocytes (Hirai et al., 2010). The activity of KMO in liver and kidney tissue decreased significantly with age (Comai, Costa, Ragazzi, Bertazzo, & Allegri, 2005). Hirai et al. (2010) found that the C‐terminal region of pig liver KMO played two important roles that the enzymatic activity requires. Second, it acts as a mitochondrial targeting signal. Furthermore, Th17 cells to self‐limit their proinflammatory activity through reducing endogenous Aryl Hydrocarbon receptor (AhR) ligand levels by the upregulation of KMO may be a new metabolic mechanism (Stephens et al., 2013). Some research also found that KMO was strongly expressed and induced in LPS and proinflammatory cytokines in human monocyte‐derived macrophages (Chiarugi, Calvani, Calvani, Meli, Traggiai, & Moroni, 2001). The following is a brief description of KMO in the treatment of non‐neurological diseases.

#### 3.2.1 Acute pancreatitis (AP)

Mole et al. (2016) found that KMO inhibitors are a new treatment for acute pancreatitis and the metabolic flux of KMO is elevated proportionately to disease severity in human AP (Skouras et al., 2016). There is compelling evidence that tryptophan metabolism has changed in a range of acutely injured environments. Therefore, the activation of KMO leads to an increase in kynurenine levels, which are critical for the development of post‐traumatic sepsis, and an increase in kynurenine and 3‐HK levels is related to the development of organ failure in acute pancreatitis (Abdel‐Magid, 2015). Some KMO inhibitors which possess differentiated binding modes and kinetics had been reported recently (Hutchinson et al., 2017; Liddle et al., 2017; Walker et al., 2017). However, human pancreatitis is highly heterogeneous, so further studies are necessary to demonstrate whether KMO inhibitors are suitable for all pancreatitis (Ray, 2016).

#### 3.2.2 Hepatocellular carcinoma (HCC)

Jin et al. (2015) detected the expression of KMO in HCC by immunohistochemistry. In vitro studies showed that KMO can absolutely regulate the proliferation, migration, and invasion of HCC cells. These results indicate that upregulation of KMO has a tumor‐promoting effect on HCC, and the mechanism may be that KMO affects the abnormal metabolism of kynurenine, leading to abnormal NAD concentration in HCC, resulting in NADH/NAD+ redox steady state destruction, thereby promoting the development of cancer, and so KMO can be used as a new prognostic marker for HCC. Badawy and Bano (2016) suggested that elevated kynurenine metabolites such as 3‐HK and 3‐HAA may be another mechanism of alcoholic aversion and anticancer effects. Gimenez‐Gomez et al. (2018) found KMO‐controlled KP can be used to alter ethanol consumption behavior while also being a regulator of drinking behavior. This may be a new direction in the treatment of liver diseases. Recently, it was reported that downregulation of KMO activity significantly inhibited cell proliferation of tumor cells and KMO may be a potential biomarker for tumor diagnosis (Chiu, Lei, Huang, Chiang, & Lin, 2019).

### 3.3 Other diseases

Stephens et al. (2013) found that inhibition of KMO activity or addition of exogenous kynurenine may be explained to a significant increase in Th17 lineage differentiation, and KMO may play the role of an endogenous AhR agonist in the background of ongoing autoimmune diseases. Therefore, stimulating the activity of KMO may be another possibility to treat immune diseases. Kubo et al. (2017) found that KMO‐deficient mice have a reduced probability of developing acute viral myocarditis, which may be a new direction for the therapy of acute viral myocarditis. However, its mechanism of action still requires further study. Oxenkrug, van der Hart, Roeser, and Summergrad (2017) found that inhibition of peripheral KMO may be a new measure for the prevention of obesity and diabetes, and KMO expression in the adipose tissue was positively correlated with increased HbAlc level (Favennec et al., 2015). Aziz, Abdel‐Salam, Al‐Obaide, Alobydi, and Al‐Humaish (2018) speculated that the identified miRNAs can regulate KMO expression by studying the 5′ and 3′ regulatory factors of the KMO gene, and in common with the alternative promoter of the 5′ regulatory region of KMO may help to develop smoking diagnosis and treatment. Zheng et al. (2019) found that inhibition of KMO activity helps to cure ischemia–reperfusion injury (IRI) after acute kidney injury (AKI). In addition, the activity of KMO as a causal factor for changes in the kidney leading to proteinuria (Korstanje et al., 2016) was increased significantly in chronic renal failure of various severity (Pawlak, Tankiewicz, Matys, & Buczko, 2003), and it may be a new research direction of kynurenine pathway.

## 4 CONCLUSION

As a vital link regulator of kynurenine metabolism, KMO plays an indispensable role in the metabolism of tryptophan and KMO inhibitor may be the potential therapy to nervous system (Raffaella, Elena, Fiamma, Pellegrini‐Giampietro, & Moroni, 2010). KMO's activity can be used to clinical diagnosis, and more and more researchers have also found that KMO is a therapeutic target for the treatment of central nervous system diseases, especially Huntington's disease, but basically focuses on the mechanism of KMO in the microglia in the brain, and less on effects of KMO in peripheral organizations (Sathyasaikumar, Breda, Schwarcz, & Giorgini, 2018). Due to the immunity and inflammation relationship of KMO, KMO may be linked to more diseases. With the development of technology, KMO knockout mice were developed and it will help us understand the biological ramifications of KMO. In addition, the level of KYN and KYNA was significantly increased in the periphery than in central nervous system (Giorgini et al., 2013). Some researchers found that peripheral KMO deficiency might be divided into at least, two patterns (Oxenkrug et al., 2017), while having fewer research mechanisms besides central nervous system diseases which requires further research.

## CONFLICT OF INTEREST

The authors declare that they do not have any conflict of interest.

## HUMAN AND ANIMAL RIGHTS

No animals or humans were used for studies that are the basis of this review.
